# Preliminary Studies about Valorization of *Acmella oleracea* Bioactive Content in Modern Dermato-Cosmetic Applications to Combat Skin Oxidative Stress

**DOI:** 10.3390/ijms25168886

**Published:** 2024-08-15

**Authors:** Delia Turcov, Adriana Trifan, Adrian Catalin Puitel, Ramona Cimpoesu, Anca Zbranca-Toporas, Claudia Maxim, Daniela Suteu, Ana Simona Barna

**Affiliations:** 1 Faculty of Medical Bioengineering, “Grigore T. Popa” University of Medicine and Pharmacy, Mihail Kogalniceanu Street No. 11-13, 700454 Iasi, Romania; delia.turcov@gmail.com (D.T.); zbranca@roderma.ro (A.Z.-T.); 2 Faculty of Pharmacy, “Grigore T. Popa” University of Medicine and Pharmacy, Universitatii Street No. 16, 700115 Iasi, Romania; adriana.trifan@umfiasi.ro; 3 Faculty of Chemical Engineering and Environmental Protection “Cristofor Simionescu”, “Gheorghe Asachi” Technical University of Iasi, Prof. Dr. docent D. Mangeron Blvd., No. 73A, 700050 Iasi, Romania; adrian-catalin.puitel@academic.tuiasi (A.C.P.); claudia.maxim@student.tuiasi.ro (C.M.); isimonabarna@yahoo.com (A.S.B.); 4 Faculty of Materials Science and Engineering, Department of Materials Science, “Gheorghe Asachi” Technical University of Iasi, Prof. Dr. docent D. Mangeron Blvd., No. 41, 700259 Iasi, Romania; ramona.cimpoesu@academic.tuiasi.ro

**Keywords:** *Acmella oleracea* alcoholic extract, vegetal biomass, dermato-cosmetic serum, skin oxidative stress

## Abstract

The development of products with skin-protective effects has been driven by the increasing incidence of skin diseases that are exacerbated by increasing pollution, urbanization, poor living, working, fatigue, dietary habits, and general treatment. The ability of antioxidants to protect the skin from oxidative stress and its effects makes them one of the most important ingredients in today’s cosmetics. This article aims first to characterize the plant extracts obtained from *Acmella oleracea* (*A. oleracea*) and then to evaluate the preliminary criteria for a new marketed product: the stability, antioxidant activity, and in vitro behavior of certain serums based on *A. oleracea* plant extract and hyaluronic acid. The extracts were obtained by liquid–solid extraction methods (maceration (M), ultrasound-assisted extraction (UEA), and a combined method between these two (UEA + M) using an aqueous solution of ethyl alcohol as the extraction solvent. The determination of the amounts of compounds with antioxidant activity highlighted the fact that the extract obtained from the whole plant of *A. oleracea* using maceration in conditions of S/L = 1:30, 20 days, and an extraction solvent percentage of 50% led to obtaining the highest amount of polyphenols (30.42 μg GAE/g), while using the combined UAE + M method under conditions of S/L = 1:30, 6 min + 20 days, and 50% extraction solvent led to obtaining the highest amount of flavonoids (32.88 mg QE/g). The tests performed on dermato-cosmetic serums based on the plant extract and multimolecular hyaluronic acid (HA) (1 HA with HMW—1.0 mDa–1.6 mDa; HA with LMW—10 kDa–200 kDa; and HA OLIGO, MW < 10 kDa) led to the conclusion that they exhibit structural stability, good shear behavior revealing a satisfactory texture, and high physical stability during storage. These results encourage the transition to in-depth testing, both microbiological and dermatological, as a final step in the consideration of a new commercial product.

## 1. Introduction

Among the plants with notable applications in the pharmaceutical and food industries, *A. oleracea*, from the *Asteraceae* family, occupies a special place due to its generous range of functional activities that may lead to new pharmacological applications. With a strong scientific background in terms of multidisciplinary research reports and already recognized as a powerful medicinal plant, *A. oleracea* (AO) seems to have not yet exhausted its potential and can be considered as part of new innovative industries, such as in the dermato-cosmetic field.

*A. oleracea* is a prominent member of the 30 species belonging to the genus *Acmella* of the *Asteraceae* family, which is distributed throughout the world in the regions of Asia, Africa, America, and also Europe. There has often been confusion and doubt in the literature due to the use of incorrect synonyms for this species, but photographs, as well as morphological, chromosomal, and molecular analyses, confirm the distinction between *A. oleracea* and similar plants such as *Spilanthes acmella* [1,2].

*A. oleracea* is mainly found in regions of South America and is used for its spicy properties and also for its sensorial effects on teeth and on other problems of the oral cavity (Table 1). Its common names are “toothache plant”, jambu, akmella, spot flower, or paracress [1].

While some *Acmella* species have some limitations (low germination capacity and seed viability [7]), *A. oleracea* benefits from specific studies on the biostimulant treatment of cultures to obtain an optimal content of active ingredients [6]. Cultivation is also a guaranteed method for ensuring a safe source that avoids contamination [10]. *A. oleracea* is an annual herb that is cultivated for many purposes, e.g., as an ornamental plant and for culinary or medicinal purposes. All its parts—flowers, leaves, and roots—are widely used and are considered very valuable in terms of the nutrients they contain (carbohydrates, fibers, proteins, lipids, minerals, and amino acids) [10,11].

As a medicinal plant, *A. oleracea* is considered to have great potential, both topically and internally [11], due to its main lipophilic N-alkylamide (N-alkamides, from isobutyla-mides), spilanthol (affinin), the most abundant active ingredient in the aerial parts, which exhibits various pharmacological activities, as shown by in vitro studies (Table 1). Alkamides are secondary metabolites with anti-inflammatory, immunomodulatory, and cannabinomimetic effects. Other bioactive compounds found in *A. oleracea* are triterpenes, coumarins, phenolic acids, stigmasterol, and myrcyl alcohol, which have various known functions in plants and in the human body, mainly anti-inflammatory, antimicrobial, antioxidant, antidiabetic, hepatoprotective, and anticarcinogenic functions [5,12,13,14,15,16].

The therapeutic properties of *A. oleracea* (using the whole plant, but mainly the aerial parts) are numerous and testify to its pharmaceutical potential: it is anti-inflammatory (based on the inhibition of chymase activity and suppression of the pro-inflammatory cytokine NO), antimicrobial (antifungal and antiprotozoal), anesthetic, antipyretic, antioxidant, insecticidal, antiseptic, immunostimulant, antitumoral [1], anti-obesity, antispasmodic, diuretic, anthelmintic, aphrodisiac [11], sialagogue, analgesic, sore throat, wound healing, antirheumatic, tonic, antimalarial [12], and hepatoprotective without causing adverse effects [17] (Table 1).

The interest of this article relates to the potential of *A. oleracea* for use in dermato-cosmetics, an industry with very dynamic research and development, for which plants are an essential support. For this purpose, the most important properties of *A. oleracea* are not only its anti-inflammatory and antioxidant effects on the treated tissue but also its anti-wrinkle effect due to a muscle-relaxant mechanism [17]. There are in vitro and in vivo studies demonstrating the anti-inflammatory effect of *A. oleracea* extract, which is thought to be related to chymase activity (a protease that promotes tissue inflammation), the reduction of NO production, and antioxidant activity [12]. These results support our interest in expanding our knowledge of this plant.

One of the most important factors in modern dermato-cosmetics is active ingredients with antioxidant activity, especially in skin diseases with a significant inflammatory component.

The current state of research on *A. oleracea* as a potential resource for valuable active ingredients presents results on: antimicrobial activity, phytochemical studies, and quantification of the alkaloids, saponins, and flavonoids of the plant [18]; antioxidant activity and nutrient content [11]; its potential functional properties, phytochemical content, and edibility as food [1]; the anti-inflammatory activity of the extracts and the main constituent, spilanthol [12]; the identification of N-alkylamides and their stability to improve scientific support for future pharmaceutical, cosmetic, and food industry use [5].

Oxidative stress is a phenomenon that has been extensively studied in recent years as a health risk factor, as it maintains or exacerbates disease throughout the body. There is evidence that oxidative stress is involved in neurological, cardiovascular, oncological, metabolic, and dermatological diseases [19,20]. Oxidative stress is an imbalance in the body’s homeostasis due to the overproduction of free radicals under the influence of factors exacerbated by modern lifestyles, with changes in diet, sleep–wake cycles, medication intake, and psychological state. In vitro studies have shown that spilanthol can abolish NO production, significantly reduce the formation of superoxide anion radicals, and increase sevenfold the activity of an important antioxidant enzyme, catalase, compared to the control [12].

Among the numerous health benefits mentioned above, these antiseptic, anesthetic, analgesic, and antioxidant effects are essential for modern, effective dermato-cosmetic formulas for a wide range of skin conditions, with increasing impact. Additionally, the antioxidant effect of other bioactive compounds potentiates specific activities and, along with the high safety level of the plant [17], recommends *A. oleracea* for further in-depth studies.

While in most studies, the entire aerial part of *A. oleracea* was examined separately [12], in this study, the following three parts of the plant were examined separately: the flowers, leaves, and stems.

In this context, this article aimed to obtain an aqueous serum based on multimolecular hyaluronic acid (HA) and containing *A. oleracea* extract as an active component. The extraction and the quantitative and qualitative characterization of the hydroalcoholic extracts of *A. oleracea* are presented, as well as a preliminary characterization of the dermato-cosmetic serum thus obtained (stability, morphology, polyphenol content, and antioxidant activity, along with in vivo studies).

## 2. Results

### 2.1. Obtaining and Characterizing the Vegetal Extracts of Acmella oleracea

**Figure 1:**
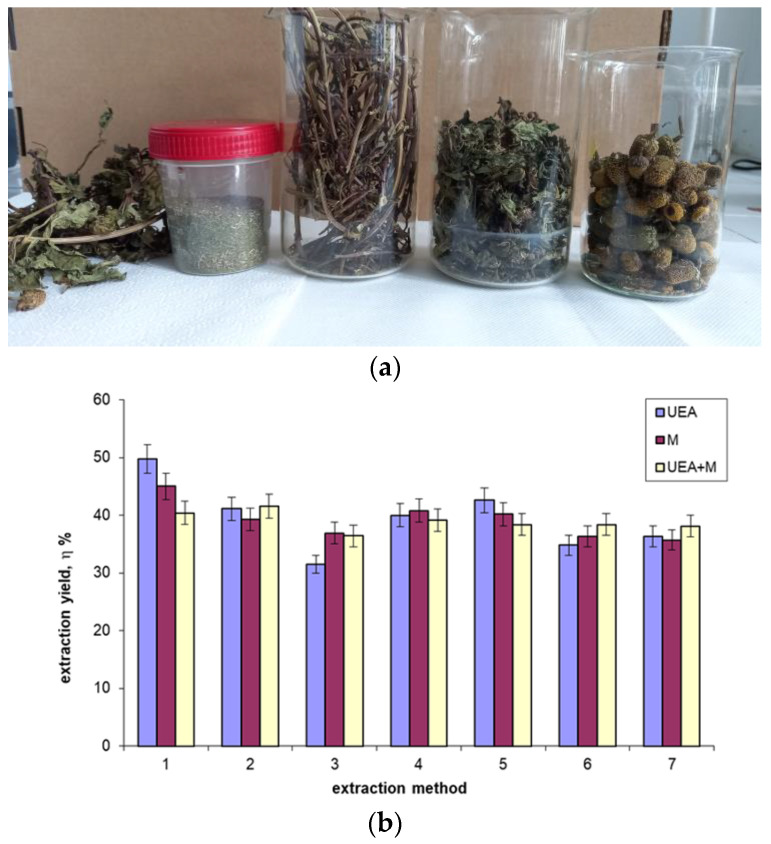

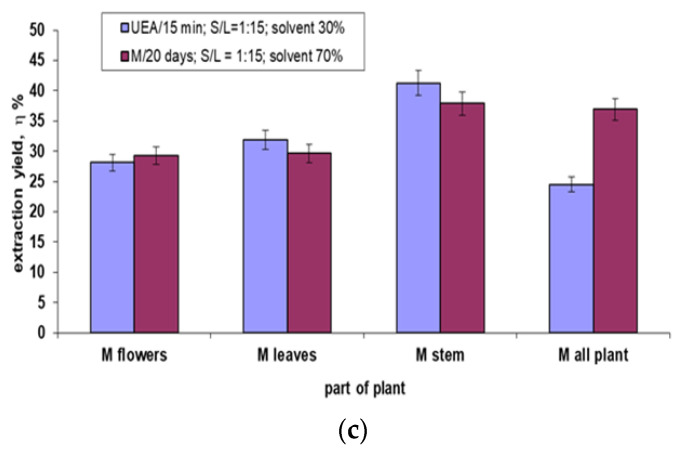
Images of *A. oleracea* plant parts, used experimentally (**a**); the obtained extraction yield in the case of using the entire plant and three extraction methods with their experimental conditions, as presented in Table 2 (**b**); the extraction yield (**c**) in the case of using different parts of the plant and two extraction methods with the experimental conditions indicated in the figure.

The flowers, leaves, stems, and entire plant (Figure 1a) were utilized for extraction in order to ascertain which part of the plant is most effective for producing plant extracts (expressed by extraction yield, but also by the amount of bioactive chemicals). Initially, we powdered and sieved the entire plant and evaluated the effectiveness of the suggested extraction techniques (Figure 1b). The extraction yield served as an expression of how well the extraction techniques worked. The outcomes are presented in Figure 1b,c.

#### 2.1.1. Total Polyphenolic Content

**Figure 2:**
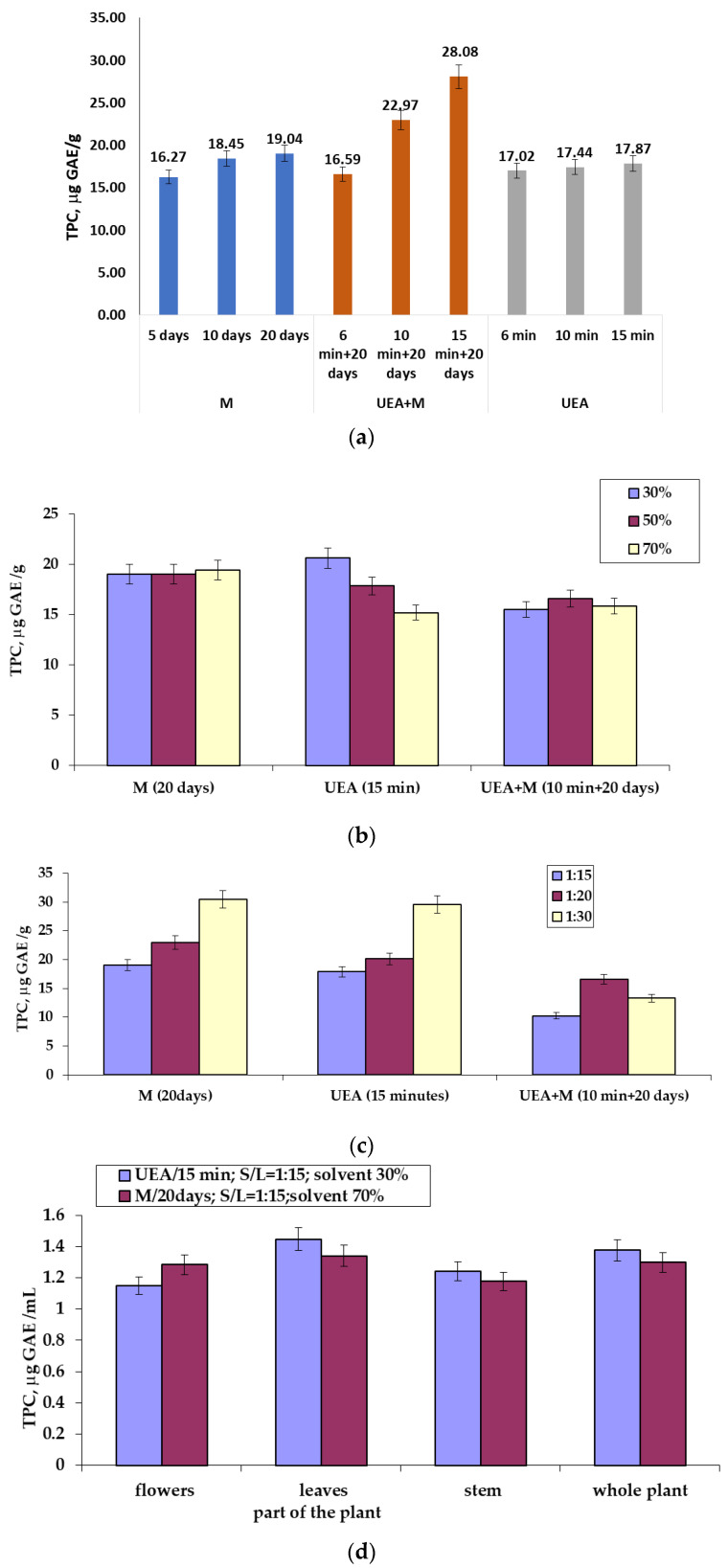
Total polyphenol content (TPC) (µg GAE/g) of *A. oleracea* extracts obtained from the whole plant (**a**–**c**) and from various parts of the plant (**d**), as a function of the extraction method used and factors influencing the process (**a**–**c**). Conditions: (**a**) S/L = 1:15; M—20 days, US—15 min, US-M—10 min + 20 days.

The determination of the amounts of polyphenols (TPC), which was performed in triplicate, was conducted on all types of extracts obtained from *A. oleracea*, the aim being to find the extract that was richest in polyphenols and to identify the extraction method and the experimental conditions that led to these results. The obtained results are presented in Figure 2.

#### 2.1.2. Total Flavonoid Content

**Figure 3:**
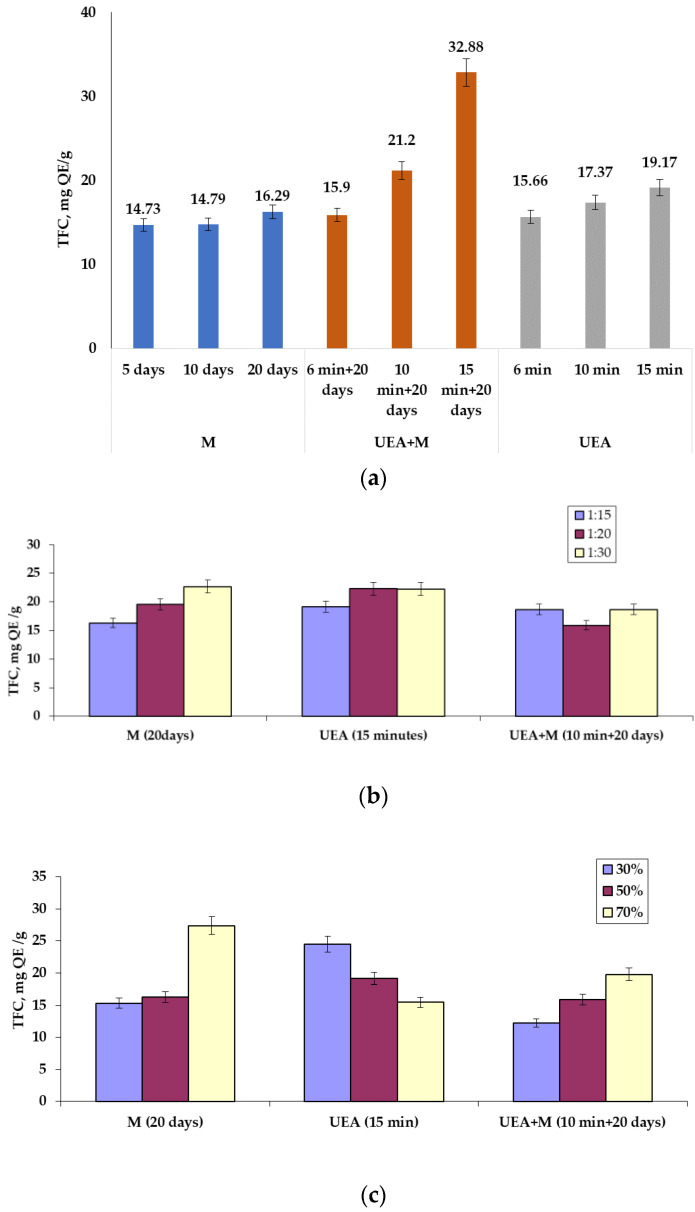

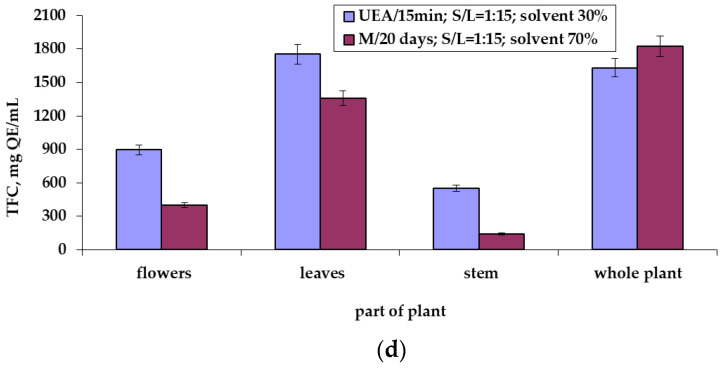
Flavonoid content (TFC in mg QE/g) of *A. oleracea* extracts obtained from the whole plant (**a**–**c**) and from various parts of the plant (**d**), depending on the extraction method used and the factors influencing the process (**a**–**c**). Conditions: (**a**) S/L = 1:15; M—20 days, US—15 min, US-M—10 min + 20 days.

Furthermore, studies were conducted in triplicate for all *A. oleracea* extract types in the case of flavonoid measurements. The results thus obtained, expressed in mg of quercetin (mg QE/g), are presented in Figure 3.

#### 2.1.3. Qualitative Characterization of Vegetal Extract 

**Figure 4:**
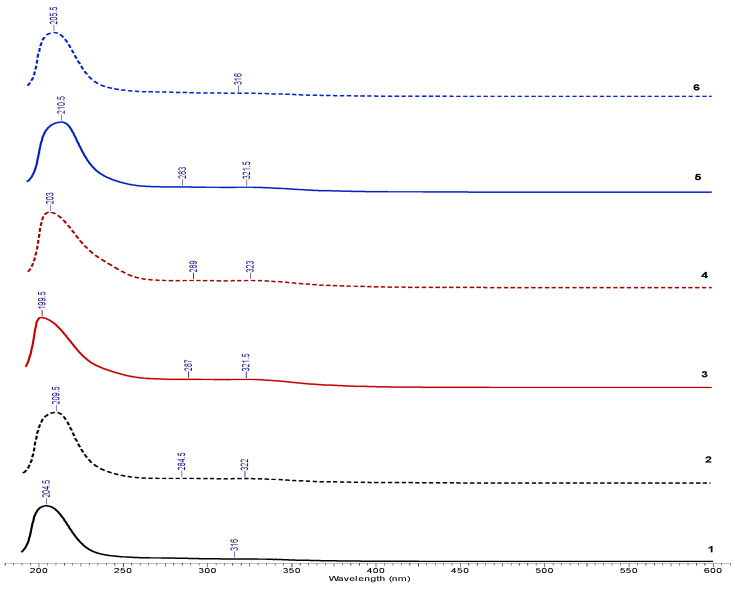
UV-Vis spectra of *A. oleracea* extracts prepared by: maceration: 1—M1 and 2—M4; sonoextraction: 3—UEA1 and 4—UEA5; and sonoextraction—maceration: 5—UEA + M 1 and 6—UEA + M2, using the whole plant for extraction.

**Figure 5:**
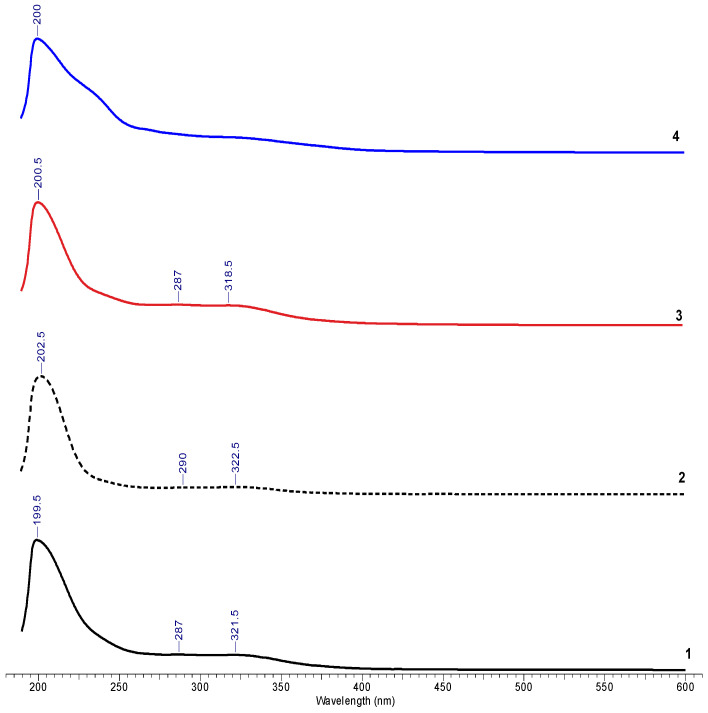
UV-Vis spectra of *A. oleracea* extracts prepared by sonoextraction using the following parts of the plant: 1—entire plant; 2—stem; 3—leaves; and 4—flowers.

Analysis of the UV-Vis spectra of hydro-alcoholic *A. oleracea* extracts can provide a set of preliminary information about the nature of the extracted compounds. Some extracts obtained after maceration, sonoextraction, and a combined method of sonoextraction + maceration were analyzed by UV-Vis spectrophotometry. The UV-Vis spectra thus obtained are illustrated in Figure 4 and Figure 5.

Absorption in the range of 200–800 nm showed the presence of a molecule that contains either pi bonds or atoms with non-bonding orbitals (such as oxygen atom or N atom). Absorption at ~200 nm shows the presence of compounds with at least one pi bond (double bond). The increase in the number of the double bonds—conjugation—shifts the absorption maxima to higher wavelengths [21]. The presence of compounds with at least one atom with non-bonding orbitals, such as the O in phenolic -OH, is indicated by the absorption around 290 nm (280–320 nm in the spectra).

### 2.2. Obtaining and Characterizing the Serum Based on A. oleracea Extract

**Figure 6:**
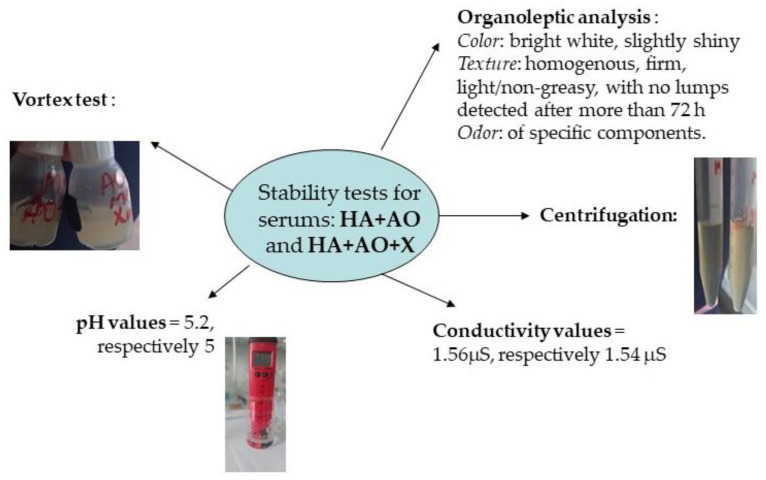
The results of the stability tests for the obtained serum, based on the vegetal extract of *A. oleracea* and hyaluronic acid.

To prepare the serums, the extract of *A. oleracea*, obtained by extraction assisted by ultrasound (using the entire plant), was used under the following conditions: 1:15 and solvent 30%, for 15 min. The results of the stability tests performed on the obtained serum based on *A. oleracea* extract and hyaluronic acid are presented in Figure 6, together with the analysis performed on the simple serum.

#### 2.2.1. Analysis of the Homogeneity and Stability of the Serums

**Figure 7:**
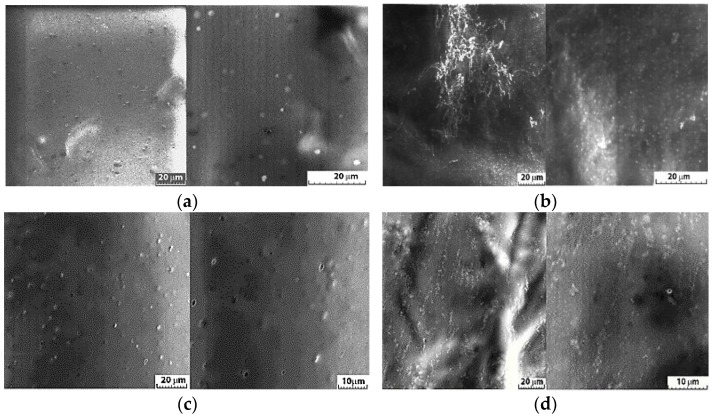
Scanning electron microscopy (SEM) of the serum without active compounds: HA-MM (**a**) and HA-XNTN (**b**), and the serum with AO extract: HA-MM-AO (**c**) and HA-XNTN-AO (**d**).

The surface morphologies and compositions were analyzed through scanning electron microscopy for characterization of the homogeneity and stability of the serums without active substances (HA-MM and HA-XNTN) and the serum with *A. oleracea* (AO) extract (HA-MM-AO and HA-XNTN-AO). Furthermore, micrographs and elemental distributions are presented for each serum sample; images obtained at 10× and 20× magnification are shown in Figure 7.

**Figure 8:**
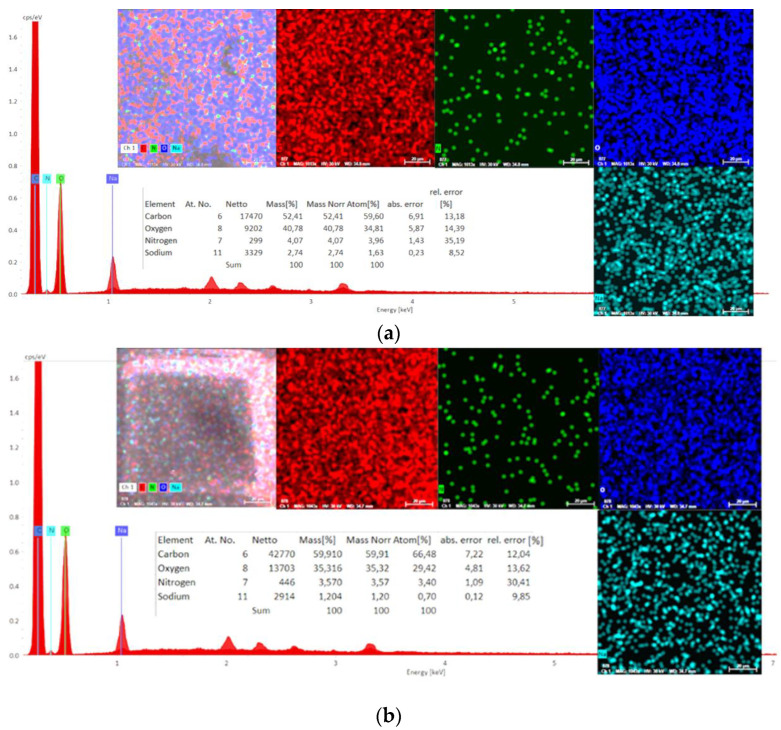
Energy-dispersive X-ray (EDX) spectra of the serum: HA-MM—without AO extract (**a**) and serum HA-MM-AO—with AO extract (**b**).

**Figure 9:**
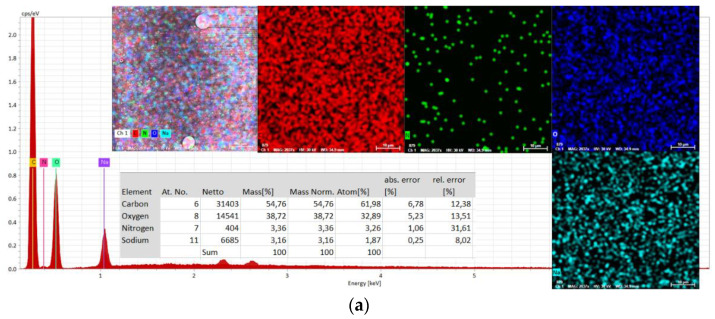

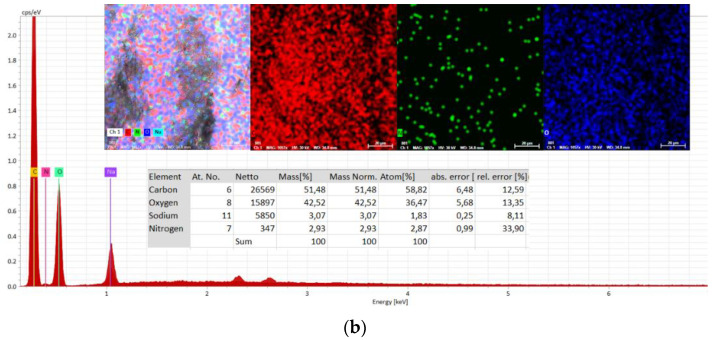
Energy-dispersive X-ray (EDX) spectra of the serum: HA-XTN—without AO extract (**a**) and serum HA-XTN-AO—with AO extract (**b**).

Also, the chemical composition of serums without and with active compounds was characterized using energy-dispersive X-ray spectroscopy (EDX). Determinations were made in three areas on the surface of the material (1 mm^2^) and an average was used (Figure 8 and Figure 9).

#### 2.2.2. Evaluation of the Total Polyphenol Content and Antioxidant Activity of Dermato-Cosmetic Serums

The results obtained are presented in Table 2. For comparison purposes, TPF was determined for the 30% ethanol *A. oleracea* extract, and the results are expressed as µg GAE/g of extract. For comparison purposes, both the DPPH and ABTS assays were performed for the 30% ethanol *A. oleracea* extract. DPPH and ABTS radical scavenging activities were expressed as micrograms of Trolox equivalents (µg TE/g extract). An enrichment of the total content of polyphenols can be observed in the case of the prepared serums. Also, the antioxidant activity of the serums increased as a result of the presence of the *A. oleracea* plant extract.

### 2.3. In Vitro Diffusion Test

**Figure 10:**
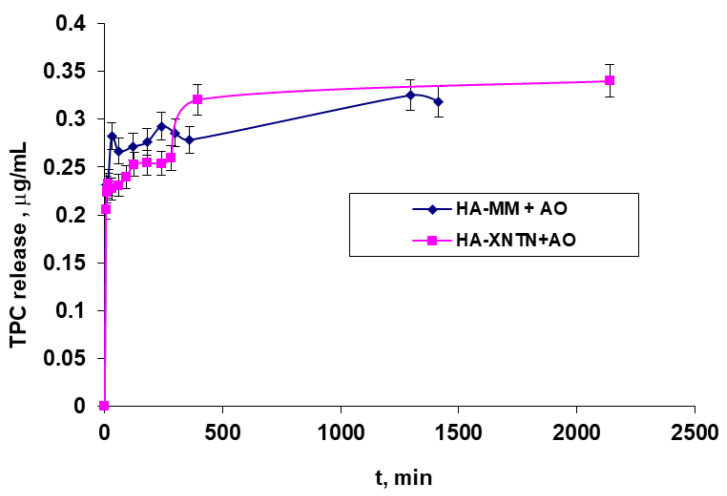
The number of polyphenols (TPF) released in the receptor compartment (5 mL), depending on time.

**Figure 11:**
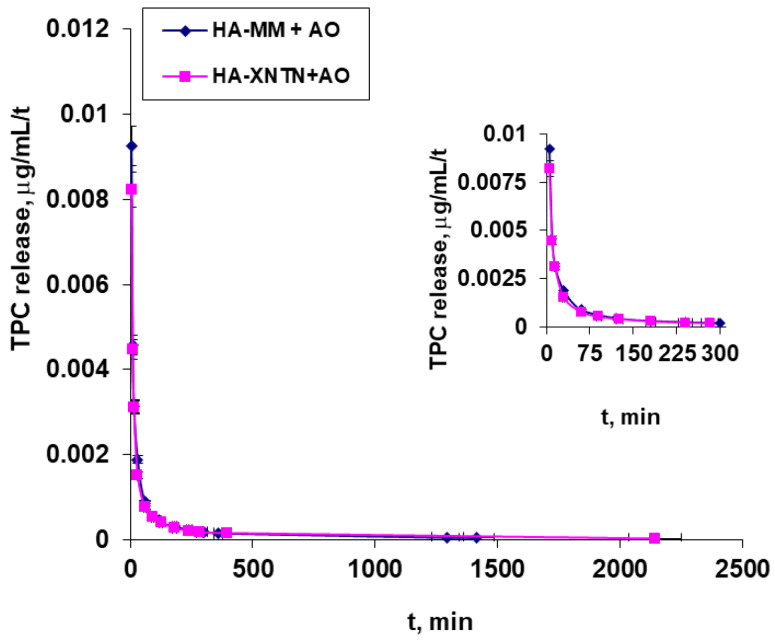
The rate of release of polyphenols, expressed in TPC μg/mL/t.

**Figure 12:**
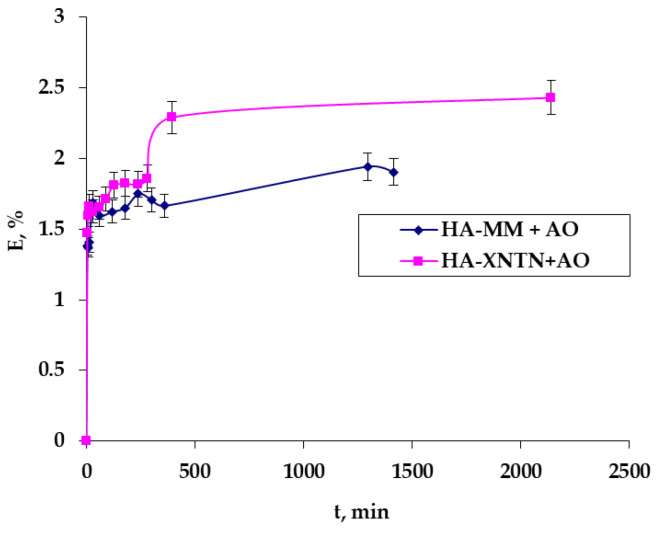
Efficiency of polyphenol permeation through the dialysis membrane.

Preliminary permeation studies were developed using the diffusion methodology with Franz cells to investigate, as appropriate, the application of serums with *A. oleracea* extract as the active ingredient by a cutaneous route and also to establish the necessary data for extending this study to real skin (chicken skin) in order to investigate the serum’s behavior in a context close to a real-life scenario. The data are presented in three different ways, each with a distinct meaning:

(1) The polyphenols released in the 5 mL of the receptor chamber, expressed in TPC, μg/mL mg, or µL, versus time (Figure 10);
(2) Polyphenol-releasing speed, expressed in TPC μg/mL/t (Figure 11); and
(3) Polyphenol permeation efficiency (Equation (1)) through a membrane (Figure 12).

E% = [(TPC release, μg)/TPC from initial sample of serum, μg] × 100.(1)

## 3. Discussion

### 3.1. Obtaining and Characterizing the Vegetal Extracts of A. oleracea

When the whole plant was used experimentally for extraction, similar results were obtained for the three extraction methods used, but it can, nevertheless, be noticed that maceration (M) and sonoextraction (UEA) led to relatively higher increased values (Figure 1b). Figure 1c shows the results, expressed as the percentage of extraction, that would be obtained if we considered as extraction methods only maceration (M, with conditions: extraction time—20 days; ratio S/L = 1:15 and solvent concentration = 70%) and sonoextraction (UEA, with conditions: extraction time—15 min; ratio S/L = 1:15, and solvent concentration = 30%), and the percentage when different parts of the plant were used as the extraction material. The results thus obtained (Figure 1c) led to the conclusion that the best results in terms of “extraction yield” were obtained in the case of extraction using the plant stem.

#### 3.1.1. Total Polyphenolic Content

The processing of the experimental data led to the results presented in Figure 2, showing the content of polyphenols as determined according to a series of physical factors that influence the extraction process. Plant extracts obtained by ultrasound-assisted extraction (UEA), maceration at room temperature (M), and a combination of UEA + M methods were used. The results of the studies showed different values, not only depending on the extraction method but also on the part of the plant used for extraction.

If the data are analyzed according to the S/L ratio, it can be observed that a greater amount of polyphenols (30.42 μg GAE/g) is obtained in the case of maceration when carried out under conditions of S/L = 1:30, a solvent concentration of 50%, and an extraction time of 20 days, followed by sonoextraction with 29.57 mg GAE/g, carried out under conditions of S/L = 1:30, a solvent concentration of 50%, and an extraction time of 15 min.

Considering the concentration of the extraction reagent as an evaluation criterion, the best results regarding the amounts of polyphenols were obtained after sonoextraction (20.63 µg GAE/g), realized under conditions of S/L = 1:15, the concentration of the solvent at 50% extraction, and an extraction time of 15 min, followed by maceration (19.46 µg GAE/g) carried out under conditions of S/L = 1:15, an extraction time of 20 days, and an extraction solvent concentration of 70%.

If the results after the extraction time are analyzed, then the best results were obtained following the application of the combined methods of UEA + M (28.08 µgGAE/g), performed under conditions of S/L = 1:20, a concentration of the extraction agent of 50%, and an extraction time of 15 min + 20 days, also followed by UEA + M (22.972 µg GAE/g), carried out under conditions of S/L = 1:20, an extraction agent concentration of 50%, and a time of extraction of 10 min + 20 days.

Analyzing all the experimental data obtained in this study, it appears that a considerable and approximately equal amount of polyphenols is obtained when using three techniques as an extraction method: maceration (30.42 µg GAE/g), carried out under conditions of S/L = 1:30, an extractant concentration of 50%, and an extraction time of 20 days; sonoextraction (29.57 µg-GAE/g), performed under conditions of S/L = 1:30, an extractant concentration of 50%, and an extraction time of 15 min; UEA + M (28.081 µg GAE/g), made under conditions of S/L = 1:20, an extractant concentration of 50%, and an extraction time of 6 min + 20 days.

Comparing the results in Figure 2a–c, when the whole plant was used for extraction, it appears that:

- Maceration (M) under conditions of S/L = 1:30, an extraction solvent concentration of 50%, and an extraction time of 20 days ensured the obtaining of 30.42 µg GAE/g polyphenols;
- Sonoextraction (UEA) under conditions of S/L = 1:30, an extraction solvent concentration of 50%, and an extraction time of 15 min ensured the obtaining of 29.57 µg GAE/g polyphenols;
- Sonoextraction combined with maceration (UEA + M) under conditions of S/L = 1:20, an extraction solvent concentration of 50%, and an extraction time of 6 min + 20 days ensured the obtaining of 28.081 µg GAE/g polyphenols.

Regarding the contents of polyphenols in the extracts obtained from different parts of the plant (Figure 2d), it follows that the methods used for extraction, namely, maceration (S/L = 1:15, extraction time of 20 days and extractant concentration of 70%) and sonoextraction (S/L = 1:15, extraction time of 15 min and extractant concentration of 30%) led to comparable results regarding the amounts of polyphenols. These results show that slightly higher amounts of polyphenols were extracted from the leaves, as follows:

- Sonoextraction: 21.69 µg GAE/g from the leaves and 20.63 µg GAE/g from the whole plant;
- Maceration: 20.099 µg GAE/g from the leaves and 19.46 µg GAE/g from the whole plant.

#### 3.1.2. Total Flavonoid Contents

The analysis of the results presented in Figure 3 was made according to three physical parameters that were considered:

- Depending on the S/L ratio, it can be observed that in general, there is an increase in the amounts of flavonoids present in the plant extracts. It can be observed that greater amounts of flavonoids at 22.68 mg QE/g were obtained following maceration carried out under conditions of S/L = 1:30, a solvent concentration of 50%, and an extraction time of 20 days, followed by two variants of sonoextraction: one with 22.28 mg QE/g, carried out under conditions of S/L = 1:20, a solvent concentration of 50%, and an extraction time of 15 min; and another variant with 22.26 mg QE/g, made under conditions of S/L = 1:30, a solvent concentration of 50%, and an extraction time of 15 min.
- Considering the concentration of the extraction reagent, it is observed that in some cases, there is an increase in the amounts of flavonoids (maceration and the combined UEA + M method) with the increase in the concentration of the extractant, and in the case of sonoextraction, there is a decrease in the amounts of flavonoids. The best results regarding the amounts of flavonoids were obtained after maceration (27.39 mg QE/g), carried out under conditions of S/L = 1:20, an extraction time of 20 days, and s 70% concentration of the extraction solvent, followed by sonoextraction (24.48 mg QE/g) achieved under conditions of S/L = 1:15, an extraction solvent concentration of 30%, and an extraction time of 15 min, with 19.8 mg QE/g achieved following the combined method UEA + M, performed under conditions of S/L = 1:15, an extraction solvent concentration of 70%, and an extraction time of 10 min + 20 days.
- Extraction time, as an analysis criterion, influences extraction methods differently. Thus, in the case of maceration and sonoextraction, no significant differences were observed in terms of the amounts of flavonoids in relation to the extraction time, while in the case of the UEA + M combination, an increase in the amounts of flavonoids was observed. The best results were obtained following the application of the combined UEA + M method (32.88 mg QE/g), performed under conditions of S/L = 1:20, an extraction solvent concentration of 50%, and an extraction time of 15 min + 20 days, followed also by the UEA+ M method (21.2 mg QE/g) performed under conditions of S/L = 1:20, an extraction solvent concentration of 50%, and an extraction time of 10 min + 20 days.

Comparing the results in Figure 3a–c, when the whole plant was used for extraction, it appears that:

- Sonoextraction combined with maceration (UEA + M) under conditions of S/L = 1:20, an extraction solvent concentration of 50%, and an extraction time of 6 min + 20 days ensured the obtaining of 32.88 mg QE/g flavonoids; the same method when carried out under conditions of S/L = 1:20, extraction solvent concentration of 50% and extraction time of 15 min + 20 days ensured the obtaining of 21.2 mg QE/g flavonoids;
- Maceration (M), under conditions of S/L = 1:15, an extraction solvent concentration of 70%, and an extraction time of 20 days, ensured the obtaining of 27.39 mg QE/g flavonoids, and, at an S/L ratio = 1:30, extraction solvent concentration of 50% and an extraction time of 20 days, ensured the obtaining of 22.68 mg QE/g flavonoids;
- Sonoextraction (UEA), under conditions of S/L = 1:15, an extraction solvent concentration of 30%, and an extraction time of 15 min, ensured the obtaining of 24.48 mg QE/g flavonoids, and, under the conditions of S/L = 1:20 and 1:30, respectively, an extraction solvent concentration of 50% and an extraction time of 15 min ensured the obtaining of 22.3 mg QE/g flavonoids.

Regarding the contents of flavonoids in the extracts obtained from different parts of the plant (Figure 3d), it transpires that two methods used for extraction, maceration (S/L = 1:15, an extraction time of 20 days, and an extractant concentration of 70%) and sonoextraction (S/L = 1:15, an extraction time of 15 min and an extractant concentration of 30%) led to results showing that a smaller amount of flavonoids was extracted from the leaves, as follows:

- Sonoextraction: 26.31 mg QE/g from the leaves and 24.48 mg QE/g from the whole plant;
- Maceration: 20.37 mg QE/g from the leaves and 27.39 mg QE/g from the whole plant.

Practically speaking, sonoextraction provides the largest amount of flavonoids from the leaves, and maceration provides the largest amount of flavonoids from the entire plant.

#### 3.1.3. Qualitative Characterization of the Vegetal Extract

The UV-VIS characterization of the *A. oleracea* plant extracts were analyzed between 200 and 400 nm, and, from Figure 4 and Figure 5, it can be seen that all spectra present a major peak at maximum wavelengths in the 199.5–210-nm range, along with a series of secondary peaks in the 318.5–323-nm range. The major peak can be attributed to spilanthol—the main component in the extracts of plants from the *Acmella* species [5,12].

### 3.2. Obtaining and Characterizing the Serum Based on Acmella oleracea Extract

The experimental data and their results (illustrated in Figure 6) show that the obtained serums, based on *A. oleracea* extract and analyzed here, have an acceptable pH for dermato-cosmetic use, and have good stability under the action of mechanical forces (shearing or vibration)—which acts on the serum, indirectly, mainly in the process of transport, storage, and use. These results qualify them for new tests, next undergoing in vitro testing regarding diffusion through the membrane.

#### 3.2.1. Analysis of the Homogeneity and Stability of the Serums

Analysis of the spectra of serums without the active compounds (Figure 8a and Figure 9a) identified C, O, Na, and N at different X-ray energies, and the spectra of the serums with the active compounds (Figure 8b and Figure 9b) revealed similar, uniform compositions with slight changes as a consequence of incorporating the specified concentrations of *A. oleracea* extract.

#### 3.2.2. Evaluation of the Total Polyphenol Contents and Antioxidant Activity of Dermato-Cosmetic Serums

From an analysis of the data in Table 2, an enrichment of the total content of polyphenols can be observed in the case of serums prepared with vegetable extract compared to plain serum. Also, the antioxidant activity of the serum increased as a result of the presence of *A. oleracea* extract. From these results, it can be deduced that the addition of *A. oleraceea* extract achieved its intended purpose of contributing to the increase in the total antioxidant activity of the dermato-cosmetic formula obtained.

#### 3.2.3. In Vitro Diffusion Test

Figure 11 shows the very high release speed occurring at the beginning of the release process, when the concentration gradient is at its highest, and how it gradually drops to its lowest value over the course of around 24 h. This behavior is the same across the two examined samples.

The reason for the relatively low release efficiency is that the active principles are primarily released from the superficial layers, those closest to the dialysis membranes, despite the system as a whole having relatively limited dynamics, particularly in the donor compartment, as well as the fact that the formulations used are relatively viscous.

Also, from an analysis of the resulting data (Figure 10, Figure 11 and Figure 12), it seems that the addition of xanthan gum in the formulation of the second serum does not significantly influence the way in which the active principle is released from it.

## 4. Materials and Methods

### 4.1. Materials

The *A. oleracea* plant comes from the personal acclimatized culture of a member of our team, from a plantation in Romania, Iasi County, Popricani village. The harvested plants (separating the aerial parts (Figure 1a)) were stored in a cool place (15–20 °C) for drying, avoiding direct sunlight. After 6–7 weeks, the plants were packed in frosted glass containers with lids to avoid UV light and humidity until use.

### 4.2. Methods

#### 4.2.1. Extraction Methods

The *A. oleracea* extract was obtained from a plant cultivated in Romania (stem, leaves, and flowers) by means of three solid–liquid extraction methods. Solid–liquid extraction is a separation technique in which the solid phase is in contact with the liquid phase (extractant) over a certain period of time, wherein certain physical operating parameters must be observed: system temperature, the solid–liquid ratio, the phase contact time, and whether an agitation system is used or not [22]. Depending on these parameters, the different techniques can be practically distinguished. For this study, we opted for fixed-temperature maceration (M), ultrasonic-assisted extraction (UAE), and a combined method of ultrasonic-assisted extraction and maceration (UAE-M). The extraction agent was 96% ethyl alcohol (procured from Chemical Company, Iasi, Romania), used as an aqueous solution at various concentrations, such as 30%, 50%, and 70%. The efficiency of these methods was monitored according to three operational physical parameters: the solid–liquid ratio (1:15; 1:20; 1:30), extractant concentration (30%, 50%, and 70%), and extraction time (chosen according to the studied extraction method) (Table 3).

The performance of these methods was evaluated by calculating the extraction yield (Equation (2)) in the case of each method, applied according to a series of physical parameters: the solid–liquid ratio, the extraction time, and the extractant concentration:

(2) $$\eta \;\%=\frac{m_{\mathrm{residue}}\cdot V_{\mathrm{extract}}}{n_{\mathrm{extract}}\cdot m_{\mathrm{solid}\;\mathrm{sample}}}\cdot 100$$

where m_residue_ is the mass of the residue obtained after evaporation to dryness, (g); V_extract_ is the volume of extract sample used for evaporation to dryness, (mL); n_extract_ is the total extract volume after liquid–solid extraction, (mL); m_solid sample_ is the mass of the plant sample used in the liquid–solid extraction process (g).

#### 4.2.2. Obtaining the Serums

The extract with the highest content of active compounds was selected for introduction into preparations of dermato-cosmetic interest.

Two types of aqueous serum have been formulated based on multimolecular hyaluronic acid (HA) (Table 4):

The first type of multimolecular HA serum (HA-MM) (from Elemental, Oradea, Romania) was obtained by dissolving 1% HA oligo and 0.5% HA LMW in *Rosa damascena* hydrosol (from Aroma Zone, France). After a 30-min rest of the mixture, 0.5% HA HMW was added with stirring for around a few minutes until the ingredients were fully incorporated, using a rotor-stator homogenizer (ESGE Zauberstab M 160 G Gourmet, Berlin, Deutschland) operating at 15,000 rpm. Finally, an active ingredient—the alcoholic extract of *A. oleracea*—in a certain amount and preservatives were added to the multimolecular HA serum.

The second type of HA–XNTN serum was formulated according to the detailed protocol of the first serum, with the addition, under mixing, of 0.4% xanthan gum for a denser texture, better permeability for the active ingredients, and higher physical stability.

#### 4.2.3. Qualitative Characterization of Vegetal Extract

Qualitative characterization was realized using the UV-VIS spectroscopy method. The UV-VIS spectra for the serum, without and with *A. oleracea* extract as the active compound, were drawn with a Jasco UV 550 VIS spectrophotometer (Jasco International Co., Ltd., Hachioji, Japan).

#### 4.2.4. Quantitative Characterization of Vegetal Extract

Quantitative characterization was performed by determining the content of total polyphenols (TPC), based on a protocol similar to the one used by the authors in previous works [23,24].

The total phenolic content was determined following previously described methods [25,26]. Briefly, 50 µL of the sample was mixed with 100 µL Folin–Ciocalteu reagent and vigorously mixed. After 3 min, 75 µL of 1% Na_2_CO_3_ solution was added, and the mixture was incubated for 2 h at room temperature in the dark. Then, the absorbance was read at 760 nm and the total phenolic content was expressed as micrograms of gallic acid equivalents (µg GAE/g).

#### 4.2.5. Serums—Physical Characterization and Stability Tests

A number of analyses that keep track of a new product’s stability are also necessary for evaluating its quality. In this regard, in accordance with the field’s quality standards, a number of analyses were performed for the prepared emulsions under specific circumstances [27,28,29], including organoleptic evaluations, pH determination, phase separation under the influence of centrifugal or vibrational force, conductivity determination, and an evaluation of homogeneity. All the samples that were analyzed were deemed to be at room temperature.

##### pH Determination

For determining the pH values of the dermato-cosmetic serums, a digital pH meter (Hanna Instruments, Indonesia) was used, following the protocol that was presented in our previous work [30].

##### Phase Separation

For centrifugation, a test was performed using 5× *g* of sample, which was introduced into a model XC-Spinplus centrifuge for 30 min at 25 °C and 3000 rpm [28]. Mechanical vibration test: This test examines the emulsions’ stability under mechanical vibration movement, which could result in formulation instability that manifests as phase separation. In a nutshell, 5 g of the sample was shaken for 30 s using a Multi-Speed Vortex MSV-3500 (Grant Instruments Ltd., Cambs, UK) vortex shaker. The test was conducted in accordance with the instructions provided in our earlier research [23,24].

##### Conductivity Measurements

The conductivity measurements were performed using a portable Hanna Instruments conductometer, with the serum samples stored at an ambient temperature of 25 °C for 20 days.

#### 4.2.6. Assessment of Total Phenolic Compounds in Dermato-Cosmetic Serums

Diluted samples of the tested serums were prepared for the analysis. Thus, dilutions were made in a mixture of ethanol: water (in a ratio of 1:1, *v*/*v*) for each dermato-cosmetic serum (100 mg/mL). The total phenolic content (TPC) was determined using a SpectroStar Nano Microplate Reader (BMG Labtech, Ortenberg, Germany) and following a previously described methodology [24].

The TPC was determined using the Folin–Ciocalteu method. Briefly, 50 µL of each sample was added to 100 µL of Folin–Ciocalteu reagent. After 3 min, 75 µL of a 1% sodium carbonate solution was added and the mixture was incubated in the dark (for 2 h, at room temperature). A blank was prepared by adding each sample (50 µL) to the Folin–Ciocalteu reagent (100 µL) without sodium carbonate. The absorbances of the sample and the blank were read at 760 nm; the absorbance of the blank was subtracted from that of the sample. The total phenolic content was expressed as micrograms of gallic acid equivalents (µg GAE/g serum).

#### 4.2.7. Antioxidant Activity Assessment of Cosmetic Serums

The antioxidant potential of the investigated samples was determined using two tests, namely, the 2,2-diphenyl-1-picrylhydrazyl (DPPH) radical scavenging assay and 2,2′-azino-bis(3-ethylbenzothiazoline) 6-sulfonic acid (ABTS) radical scavenging assay, respectively, following previously described methodologies [24].

##### DPPH Radical Scavenging Assay

First, 50 µL of the sample was added to 150 µL of 2,2-diphenyl-1-picrylhydrazyl (DPPH) 0.004% methanol solution. After 30 min of incubation, the absorbance was read at 517 nm. DPPH radical scavenging activity was expressed as micrograms of Trolox equivalent (µg TE/g serum).

##### ABTS Radical Scavenging Assay

ABTS•+ was generated by mixing 7 mM of 2,2′-azino-bis(3-ethylbenzothiazoline) 6-sulfonic acid (ABTS) solution with 2.45 mM of potassium persulfate (1:1, *v*/*v*). The mixture was allowed to stand for 12–16 h in the dark at room temperature. At the beginning of the assay, the ABTS solution was diluted with methanol to reach an absorbance of 0.700 ± 0.02 at 734 nm. Then, 30 µL of the sample was added to 200 µL of ABTS solution and the absorbance was read at 734 nm after 30 min of incubation. The ABTS radical scavenging activity was expressed as micrograms of Trolox equivalents (µg TE/g serum).

#### 4.2.8. Analysis of the Homogeneity and Stability of the Serums

The homogeneity and stability of the serums during the storage period were studied using two analysis methods: scanning electronic microscopy (SEM) and energy-dispersive spectroscopy (EDX), according to a protocol previously described in our earlier studies [31]. For this purpose, the samples were maintained in a vacuum for 24 h. Images were obtained with a secondary electron (Ses) detector (WD 15.5 mm, 30 kV, HV) from a scanning electron microscope (SEM), the Vega Tescan-LMHII (Tescan Orsay Holding, Brno-Kohoutovice, Czech Republic). An automatic mode was used for the determination of chemical composition with energy-dispersive spectroscopy equipment from Bruker Nano GmbH Berlin, Germany. For mapping the distribution of the elements, the Esprit 2.2 software was used in automatic mode.

#### 4.2.9. In Vitro Evaluation of the Serums

In order to investigate the application of serums containing *A. oleracea* extract as the active ingredient by a cutaneous route, as appropriately as possible, and to establish the necessary data for extending this study to real skin, preliminary permeation studies were developed using a Franz cell equipped with chicken skin. This natural membrane was prepared beforehand by degreasing it with a prepeeling solution with glycolic, salicylic, and lactobionic acid (Mesoestetic, Madrid, Spain).

The donor compartment, in which a precise amount of serum (0.811 g) weighed at room temperature was introduced, and the receiver chamber, in which a volume of approximately 5 mL of double-distilled water was provided, are separated by chicken skin. At predefined intervals (from 5 min to 24 h), samples of 150 µL of emulsion were obtained from the receptor chamber (which was necessary to determine the amounts of polyphenols that passed through the membrane), and the removed volume was replenished with the exact same volume of distilled water so that the volume in the compartment remained constant. The working protocol took into account the findings from our earlier investigations [31,32,33], as well as those from the literature [34,35].

## 5. Conclusions

The purpose of the experimental procedures included in this article is to build on biotechnological and chemical processes to test certain methods and parameters, in order to prove their feasibility in terms of the applicability of the proposed protocols.

The results allowed us to select the extract with the best efficiency and the highest content of active compounds for introduction into preparations of dermato-cosmetic interest.

The obtained results show that using the extraction method of sonoextraction combined with maceration (US + M), under conditions of an S/L ratio = 1:20, a concentration of the extraction solvent of 50%, and an extraction time of 6 min + 20 days, the maximum amounts of polyphenols (28.081 mg QAE/g) and flavonoids (32.88 mg QE/g) can also be obtained. If only the stipulations regarding the amounts of polyphenols are taken into account, then effective results are obtained by maceration for 20 days, using ethanol as an extraction agent in the form of an aqueous solution of 50% concentration and a solid/liquid ratio of 1:30. If only the stipulations regarding the quantity of flavonoids are taken into account, then effective results are obtained by using the UEA + M combined method, carried out under conditions of S/L = 1:20, an extraction solvent concentration of 50%, and an extraction time of 6 min + 20 days. The results obtained for the determination of the two types of active compounds allow for the choice of the method and working conditions, depending on the purpose for which the sample is intended and the economic conditions available at a given time.

The whole plant of *A. oleracea* appears to present the best results in the extraction and that is why it was selected to be introduced into preparations of dermato-cosmetic interest, namely, serums with HA in two different formulas, to investigate their greater penetration into the dermis.

The other bioactive compounds of the plant potentiate specific activities, such as its antioxidant effect, which recommends *A. oleracea* for in-depth further studies.

Both the ingredients with high antioxidant potential and the physical properties of the serum increase the success rate of the product, this could be as a pleasant component of the daily routine that, entered into the habit of daily skin care, also ensure the active ingredients’ with a real chances of protection. 

The results of the in vitro tests presented in this work leave open the way to deeper studies of this extract and the serums obtained with it as a biologically active compound and underline the fact that these serums could be transformed into new dermato-cosmetic formulations with the perspective of combating oxidative stress on the skin.

The goal of emphasizing the diffusion system’s functionality in the context of employing a new serum has been accomplished, and this study serves as a crucial first step for future research.

## Figures and Tables

**Table 1:** Investigation of the different species from the *Acmella* genus (*Asteraceae* family) [3,4,5,6,7,8,9].

Plant	Extraction/Isolation Methods	Solvent	Purification/Characterization Methods of Active Compounds (N-Alkylamides)	Active Compounds/Biological Effects
*Spilanthes acmella*	Solvent extraction Maceration Soxhlet extraction Super-critical fluid extraction	Chloroform, ethanol, butanol, hexane, and methanol	Normal-phase chromatography Solvent partitioning Preparative HPLC HPTLC/TLC HPLC FTIR LCMS HRMS NMR GCMS CPC	Antioxidant, anti-proliferative, vasorelaxant, anti-inflammatory, enzyme inhibition and cytotoxicity, and antiwrinkle (muscle relaxant)
*Acmella uliginosa*		Methanol, dichloromethane, and distilled water		Antioxidant and anti-microbial
*Acmella oleracea*		Methanol and distilled water		Antioxidant, gastro-protective, antiproliferative, immunomodulatory, diuretic, anti-microbial, enzyme inhibition and cytotoxicity, and antiwrinkle
*Spilanthes paniculata*		Petroleum ether, ethyl acetate, and ethanol		Anti-proliferative and diuretic
*Spilanthes mauritiana*		Hexane, dichloromethane, acetone, and methanol		Anti-microbial
*Acmella ciliata*		Methanol		Anti-microbial, antipyretic, antioxidant, and vasorelaxant

**Table 2:** TPC and antioxidant activity of *A. oleracea* extract and cosmetic serums.

Sample	TPC (µg GAE/g Serum)	DPPH (µg TE/g Serum)	ABTS (µg TE/g Serum)
AO	1057.52 ± 34.04	737.96 ± 20.25	2387.67 ± 31.34
HA + MM	16.25 ± 0.74	58.52 ± 1.21	352.64 ± 12.50
HA-MM + AO	131.01 ± 8.07	96.48 ± 3.01	992.53 ± 40.69
HA + XNTN	16.78 ± 0.89	52.04 ± 1.52	284.91 ± 7.63
HA + XNTN + AO	121.69 ± 4.21	83.52 ± 5.69	740.31 ± 18.14

Notes: Data are presented as mean ± standard deviation (SD) of three determinations. Abbreviations: ABTS—2,2′-azino-bis(3-ethylbenzothiazoline) 6-sulfonic acid; DPPH—1,1-diphenyl-2-picrylhydrazyl; GAE, gallic acid equivalents; TE—Trolox equivalents; TPC—total phenolic content; AO- *A. oleracea* extract; HA + MM—simple serum based on multimolecular hyaluronic acid (HA) (MM); HA-MM + AO—serum based on AO and simple serum based on multimolecular HA (HA-MM); HA + XNTN—serum based on HA and xanthan gum (XNTN); HA + XNTN + AO—serum based on AO, hyaluronic acid (HA), and xanthan gum.

**Table 3:** The extraction methods used and the practical values of monitoring parameters.

Extraction Method	Extraction Time	Solid–Liquid Ratio (S/L)	Solvent Concentration	Sample Notation
UEA	15 min	1:15	30%	UEA1
			50%	UEA2
			70%	UEA3
	15 min	1:20	50%	UEA4
	15 min	1:30		UEA5
	6 min			UEA6
	10 min			UEA7
M	20 days	1:15	30%	M1
	20 days		50%	M2
	20 days		70%	M3
	20 days	1:20	50%	M4
	20 days	1:30		M5
	5 days	1:15		M6
	10 days			M7
UEA + M	10 min + 20 days	1:20	30%	UEA-M1
			50%	UEA-M2
			70%	UEA-M3
	15 min + 20 days		50%	UEA-M4
	6 min + 20 days			UEA-M5
	10 min + 20 days	1:30		UEA-M6
	10 min + 20 days	1:15		UEA-M7

**Table 4:** Chemical composition of the prepared serums based on *A. oleracea* extract and hyaluronic acid (HA).

Name of Raw Material According to the International Nomenclature of Cosmetic Ingredients (INCI)	%
Multimolecular HA serum “HA-MM”	
Sodium hyaluronate, HA with HMW—1.0~1.6 mDa	0.5
Sodium hyaluronate, HA with LMW—10~200 kDa	0.5
Sodium hyaluronate, HA OLIGO, MW < 10 kDa	1
Hydrosol *Rosa Damascena* (from Aroma Zone, France)	97.4
Conservant Cosgard (benzyl alcohol and dehydroacetic acid) (from Aroma Zone, France)	0.6
Serum with HA-MM and AO extract	
3.75 g serum HA MM formula + 25 mL pure *A. oleracea* extract	
HA serum with xanthan “HA-XNTN”	
Sodium hyaluronate, HA with HMW—1.0~1.6 mDa	0.5
Sodium hyaluronate, HA with LMW—10~200 kDa	0.5
Sodium hyaluronate, HA OLIGO, MW: <10 kDa	1
Hydrosol *Rosa Damascena* (from Aroma Zone, France)	97
Conservant Cosgard (benzyl alcohol and dehydroacetic acid)	0.6
Xanthan gum (from Aroma Zone, France)	0.4
HA and xanthan serum (HA-XNTN) + AO extract	
3.75 g serum “HA + XNTN” + 25 mL pure *A. oleracea* extract	

## Data Availability

The original contributions presented in the study are included in the article, further inquiries can be directed to the corresponding author.

## References

[1] Uthpala T.G.G., Navaratne S.B. (2020). *Acmella oleracea* plant; identification, applications and use as an emerging food source—Review. Food Rev. Int..

[2] Panyadee P., Inta A. (2022). Taxonomy and ethnobotany of *Acmella (Asteraceae)* in Thailand. Biodiversitas.

[3] Sharma R., Arumugam N. (2021). N-alkylamides of *Spilanthes* (syn: *Acmella*): Structure, purification, characterization, biological activities and applications—A review. Future Foods.

[4] Sharma R., Karunambigai A., Gupta S. (2022). Arumugam N, Evaluation of biologically active secondary metabolites isolated from the toothace plant *Acmella ciliata* (*Asteracee*). Adv. Tradit. Med..

[5] Savic S., Petrovic S., Savic S., Cekic N. (2021). Identification and photostability of N-alkylamides from *Acmella oleracea* extract. J. Pharm. Biomed. Anal..

[6] Suteu D., Rusu L., Zaharia C., Badeanu M., Daraban G.M. (2020). Challenge of Utilization Vegetal Extracts as Natural Plant Protection Products. Appl. Sci..

[7] Rani A.S., Sana H., Sulakshana G., Shravya E., Keerti M. (2019). *Spilanthes* acmella—An important medicinal plant. Int. J. Minor Fruits Med. Aromat. Plants.

[8] Prachayasittikul V., Prachayasittikul S., Ruchirawat S. (2013). High therapeutic potential of *Spilanthes Acmella*: A review. EXCLI J..

[9] Dias A.M.A., da Silva A.C.S., Botelho J.R.S., Junior R.N.C., de Sousa H.C. (2017). Temperature and density effects of the scCO_2_ extraction of spilanthol from *Spilanthes acmella* flowers. J. Supercrit. Fluids.

[10] Neves D.A., Da Silva Oliveira W., Petrarca M.H., Rodrigues M.I., Teixeira Godoy H. (2021). A multivariate approach to overcome chlorophyll interferences in the determination of polycyclic aromatic hydrocarbons in jambú (*Acmella oleracea* (L.) R.K. Jansen). J. Food Compos. Analysis..

[11] Lalthanpuii P.B., Hruaitluangi L., Sailo N., Lalremsanga H.T., Lalchhandama K. (2017). Nutritive value and antioxidant activity of *Acmella oleracea* (*Asteraceae*), a variety grown in Mizoram, India. Int. J. Phytopharm..

[12] Stein R., Berger M., Santana de Cecco B., Peixoto Mallmann L., Barros Terraciano P., Driemeier D., Rodrigues E., Beys-da-Silva W.O., Konrath E.L. (2021). Chymase inhibition: A key factor in the anti-inflammatory activity of ethanolic extracts and spilanthol isolated from *Acmella oleracea*. J. Ethnopharmacol..

[13] Ghosh S. (2020). Triterpenoids: Structural diversity, biosynthetic pathway, and bioactivity. Stud. Nat. Prod. Chem..

[14] Rohman N., Ardiansah B., Wukirsari T., Judeh Z. (2024). Recent Trends in the Synthesis and Bioactivity of Coumarin, Coumarin–Chalcone, and Coumarin–Triazole Molecular Hybrids. Molecules.

[15] Kumar N., Goel N. (2019). Phenolic acids: Natural versatile molecules with promising therapeutic applications. Biotechnol. Rep..

[16] Jasemi S.V., Khazaei H., Morovati M.R., Joshi T., Aneva I.Y., Farzaei M.H., Echeverría J. (2024). Phytochemicals as treatment for allergic asthma: Therapeutic effects and mechanisms of action. Phytomedicine.

[17] Benelli G., Pavela R., Drenaggi E., Maggi F. (2019). Insecticidal efficacy of the essential oil of jambú (*Acmella oleracea* (L.) R.K. Jansen) cultivated in central Italy against filariasis mosquito vectors, houseflies and moth pests. J. Ethnopharmacol..

[18] Mamidala E., Prasad G.R. (2013). Phytochemical and Antimicrobial Activity of *Acmella paniculata* Plant Extracts. J. Bio Innov..

[19] Dasgupta A., Klein K., Dasgupta A., Klein K. (2014). Combating Oxidative Stress with a Healthy Lifestyle. Antioxidants in Food, Vitamins and Supplements.

[20] Reuter S., Gupta S.C., Chaturvedi M.M., Agarwal B.B. (2010). Oxidative stress, inflamation and cancer: How are they linked. Free Radic. Biol. Med..

[21] https://chem.libretexts.org/Bookshelves/Physical_and_Theoretical_Chemistry_Textbook_Maps/Supplemental_Modules_(Physical_and_Theoretical_Chemistry)/Spectroscopy/Electronic_Spectroscopy/Electronic_Spectroscopy_Basics/What_Causes_Molecules_to_Absorb_UV_and_Visible_Light.

[22] Cascaval D., Oniscu C., Galaction A.I. (2004). Biochemical Engineering and Biotechnology. 3. Separation Processes.

[23] Turcov D., Barna A.S., Blaga A.C., Ibanescu C., Danu M., Trifan A., Zbranca A., Suteu D. (2022). Dermatocosmetic Emulsions Based on Resveratrol, Ferulic Acid and Saffron (Crocus sativus) Extract to Combat Skin Oxidative Stress-Trigger Factor of Some Potential Malignant Effects: Stability Studies and Rheological Properties. Pharmaceutics.

[24] Turcov D., Barna A.S., Trifan A., Blaga A.C., Tanasa A.M., Suteu D. (2022). Antioxidants from *Galium verum* as Ingredients for the Design of New Dermatocosmetic Products. Plants.

[25] Grochowski D.M., Uysal S., Aktumsek A., Granica S., Zengin G., Ceylan R., Locatelli M., Tomczyk M. (2017). In vitro enzyme inhibitory properties, antioxidant activities, and phytochemical profile of *Potentilla thuringiaca*. Phytochem. Lett..

[26] Luca S.V., Kulinowski L., Ciobanu C., Zengin G., Czerwinska M.E., Granica S., Xiao J., Skalicka-Wozniak K., Trifan A. (2022). Phytochemical and multi-biological characterization of two Cynara scolymus L. varieties: A glance into their potential large scale cultivation and valorization as bio-functional ingredients. Ind. Crops Prod..

[27] Sinha V.R., Mumar A. (2002). Multiple Emulsions: An overview of Formulation, Characterization, Stability and Applications. Indian J. Pharm. Sci..

[28] Kim K.M., Oh H.M., Lee J.H. (2020). Controlling the emulsion stability of cosmetics through shear mixing process. Korea-Aust. Rheol. J..

[29] Mahmood T., Akhtar N. (2013). Stability of a cosmetic multiple emulsion loaded with green tea extract. Sci. World J..

[30] Lukić M., Pantelić I., Savić S.D. (2021). Towards Optimal pH of the Skin and Topical Formulations: From the Current State of the Art to Tailored Products. Cosmetics.

[31] Barna A.S., Maxim C., Trifan A., Blaga A.C., Cimpoesu R., Turcov D., Suteu D. (2023). Preliminary Approaches to Cosmeceuticals Emulsions Based on N-ProlylPalmitoyl Tripeptide-56 Acetat-Bakuchiol Complex Intended to Combat Skin Oxidative Stress. J. Mol. Sci..

[32] Turcov D., Peptu A.C., Zbranca A., Suteu D. (2023). In vitro evaluation of the dermatocosmetic emulsions based on saffron (*crocus sativus*) alchoolic extracts. Bull. Inst. Politeh. Iasi.

[33] Turcov D., Peptu A.C., Barna A.S., Zbranca A., Suteu D. In vitro evaluation of the dermatocosmetic emulsions based on Lady’s Bedstraw (*Galium verum*) alchoolic extracts. Proceedings of the 10th IEEE International Conference on E-Health and Bioengineering—EHB 2022, Grigore T. Popa University of Medicine and Pharmacy.

[34] Bujor A., Ochiuz L., Sha`at M., Stoleriu I., Stamate Iliuta M., Luca S.V., Miron A. (2020). Chemical, antioxidant and In vitro permeation and penetration studies of extracts obtained from *Viburnum opulus* and *Crataegus pentagyna*. Farmacia.

[35] Abla M.J., Banga A.K. (2012). Quantification of skin penetration of antioxidants of varying lipophilicity. Int. J. Cosmetic Sci..

